# Editorial: The impact of GIP/GIPR on metabolic diseases: how the field is evolving

**DOI:** 10.3389/fendo.2025.1710843

**Published:** 2025-10-14

**Authors:** Kirk Habegger, Alejandra Tomas, Roger Chen, Charles Harris, William Roell, Tamer Coskun

**Affiliations:** ^1^ Comprehensive Diabetes Center and Department of Medicine - Division of Endocrinology, Diabetes and Metabolism, University of Alabama at Birmingham, Birmingham, AL, United States; ^2^ Section of Cell Biology and Functional Genomics, Division of Diabetes, Endocrinology, and Metabolism, Department of Metabolism, Digestion and Reproduction, Imperial College London, London, United Kingdom; ^3^ Department of Endocrinology, St Vincent’s Hospital, University of New South Wales, Darlinghurst, NSW, Australia; ^4^ Lilly Research Laboratories, Indianapolis, IN, United States

**Keywords:** GIP - glucose-dependent insulinotropic peptide, GIPR, intretin-based therapy, obesity, type-2 diabetes (T2DM)

The incretin effect describes the enhancement of glucose-dependent insulin secretion when glucose is delivered orally as compared to intravenously (1). This is driven primarily by two gut-derived peptide hormones, glucose-dependent insulinotropic polypeptide (GIP) and glucagon-like peptide-1 (GLP-1) (2, 3). GLP-1 and GIP are released in response to food intake by intestinal L cells and K cells, respectively (4–6). These hormones are fundamental in glucose homeostasis accounting for 50-70% of the insulin response to oral glucose with GIP as the predominant regulator in healthy individuals (7), primarily by enhancing glucose-stimulated insulin secretion from pancreatic β-cells. Beyond glucose regulation, GIP and GLP-1 play crucial roles in appetite regulation and body weight (6, 8). For decades, research and development of GLP-1 receptor (GLP-1R) agonists has proven clinically beneficial. However, more recent preclinical and clinical exploration of GIP and GIP receptor (GIPR) pharmacology has resulted in newfound interest in GIPR as a target for the treatment of type 2 diabetes and obesity.

Although discovered before GLP-1, GIP was originally named “gastric inhibitory polypeptide” due to high-dose effects on gastric motility and acid secretion (9). Its primary physiological role was later identified to enhance glucose-dependent insulinotropic action, and thus it was renamed “glucose-dependent insulinotropic polypeptide”. GIP is a 42-amino acid peptide hormone (10). Similar to GLP-1, GIP has a short half-life (7 minutes) and is inactivated by DPP-4 into GIP(3-42) (11). GIP signals via a GPCR, the GIP receptor (GIPR) (12). The GIPR gene is expressed in various peripheral tissues, including pancreatic β-cells, adipose tissue, stomach, bone, and heart, as well as several brain regions including the olfactory bulb, cerebral cortex, hippocampus, and brainstem (6). Like GLP-1R, GIPR activation in pancreatic β-cells potently stimulates glucose-dependent insulin secretion. GIPR also exerts broad extra-pancreatic actions including regulation of bone, vasculature, heart rate, arterial blood pressure (6), capillary perfusion and triacylglycerol deposition in adipose tissue (13). Importantly GIPR agonism has demonstrated weight-independent improvements in insulin sensitivity (14) as well as improving adipose tissue lipid metabolism (13). Additionally, GIPR activation contributes to body weight reduction through centrally mediated appetite suppression and may alleviate drug-induced nausea (15).

While harnessing incretin signaling for the treatment of T2D and obesity has focused on GLP-1R monoagonism, more recent findings with GIPR and GLP-1R co-agonism identifies GIPR as an important therapeutic target as well. Preclinical and clinical studies have demonstrated combining GIPR and GLP-1R agonism in a single molecule confers superior glucose and weight lowering compared to GLP-1R monoagonism (16). Clinical studies with a GIP/GLP-1 receptor agonist have demonstrated increased insulin sensitivity, insulin secretion, and fat oxidation, and furthermore reduced calorie intake compared with selective GLP-1 receptor agonist (17–19).

Any discussion of this Research Topic would be incomplete without mentioning the paradoxical evidence that both activating and inhibiting the GIP receptor can lead to beneficial effects in the treatment of obesity and T2D. In contrast to the wealth of experimental physiology and pharmacology identifying the benefits of GIPR agonism, genetic evidence has suggested that GIPR antagonism may be metabolically favorable as well including potential effects on body weight (20). This has led to pharmaceutical development of GIPR antagonists either on their own or as unimolecular multiagonists (coupled to GLP-1RAs). Consistent with the metabolic benefits associated with unimolecular GIP/GLP-1R co-agonists, GIP-overexpression and adipocyte-specific GIPR overexpression protects mice from diet-induced obesity (21). Conversely, inhibition of GIPR signaling imparts clear benefits in obesity and T2D models. *Gipr* deletion and pharmacological GIPR antagonism in mice provide protection from diet-induced obesity and glucose intolerance (22, 23). While mechanisms underlying this paradox are still under investigation, ultimately, this situation highlights the multifaceted nature of GIP biology, where both increasing and decreasing GIPR signaling has demonstrated therapeutic advantages depending on the specific metabolic context.

Articles in this special edition expand upon these themes and detail the impact that GIP/GIPR based therapies have had on the treatment of metabolic diseases. Corrao et al. review and summarize cardiometabolic benefits of the dual GIPR and GLP-1R agonist, tirzepatide, in clinical trials including weight loss, glucose reduction, blood pressure, and lipid levels. Douros et al. review the physiology of common adverse events observed with GLP1-R agonists such as nausea and how GIPR modulates these neural pathways. Koefoed-Hansen et al. review the history and current data supporting GIPR antagonism in the treatment of metabolic disease and describe some of the current GIPR antagonists in clinical development. James-Okoro et al. describe a novel mechanism in which GIPR activation may mediate neural pathways indirectly by acting on glial oligodendrocytes. Liu describe ethical and social challenges in a post-incretin world. Peiber et al. describe counterregulatory hormonal response in people with T2D treated with dual GIP and GLP-1 receptor agonist, tirzepatide versus placebo during a hypoglycemic clamp procedure.

The advent of GLP-1R agonists has changed the treatment landscape for individuals with T2D and obesity. This field has again been revolutionized by the inclusion of GIPR agonism in the form of single-molecule co-agonism. The efficacy of these treatments is without question; and additional hormone receptor affinity (e.g. glucagon and amylin receptors) may enhance treatments in this already efficacious class. Finally, the biology of GIPR agonism vs antagonism is still an open debate that will likely influence the application of these therapeutics in the future.
